# Absolute Stenosis Measures of Renal Artery Independently Influence Kidney Perfusion in Contrast-Enhanced Multidetector Computed Tomography

**DOI:** 10.3390/jcm13175022

**Published:** 2024-08-25

**Authors:** Arkadiusz Lubas, Arkadiusz Zegadło, Emilia Frankowska, Ewelina Jędrych, Tymoteusz Lubas, Anna Grzywacz, Ksymena Leśniak, Stanisław Niemczyk

**Affiliations:** 1 Department of Internal Diseases Nephrology and Dialysis, Military Institute of Medicine—National Research Institute, 04-141 Warsaw, Polandsniemczyk@wim.mil.pl (S.N.); 2 Department of Radiology, Military Institute of Medicine—National Research Institute, 04-141 Warsaw, Poland; 3 Faculty of Medicine, Medical University of Gdańsk, 80-210 Gdańsk, Poland

**Keywords:** renal artery stenosis, kidney perfusion, computed tomography, Doppler ultrasound

## Abstract

**Background:** A renal artery lumen reduction of ≥60% indicates hemodynamically significant stenosis and is one of the main criteria for invasive revascularization. We hypothesize that direct parameters describing renal artery stenosis (RAS) could better correlate with renal blood flow and improve the criterion for revascularization. This study aimed to investigate RAS parameters independently associated with renal blood flow estimated in contrast-enhanced multidetector computed tomography (CE-MDCT). **Methods:** Ultrasound Doppler dynamic renal cortical perfusion (dRCP), CE-MDCT with cortical blood flow (CBF), and RAS assessment in the form of cross-sectional area reduction (CSAR), maximal diameter reduction (MaxDR), mean diameter (MeD), and minimal diameter (MinD) were investigated. **Results:** CBF correlated with CSAR (r = −0.422, *p* = 0.003), MeD (r = 0.344, *p* = 0.005) and MinD (r = 0.348, *p*= 0.005), whereas RCP correlated only with MeD (r = 0.357, *p* = 0.005) and MinD (r = 0.427, *p*< 0.001). In multivariable regression, only MeD was independently associated with CBF (R^2^ = 0.179; *p* < 0.001), and MeD < 3.5 mm substantially indicated CBF < 175 mL/100 g/min in ROC analysis. **Conclusions:** The directly measured mean diameter of RAS is independently associated with renal cortex blood flow and is probably a more appropriate parameter for the invasive RAS treatment criterion.

## 1. Introduction

Hemodynamically significant renal artery stenosis (RAS) can contribute to resistant hypertension and worsening kidney function, with incidents of malignant hypertension and acute cardiorenal syndrome expressed as sudden unexplained pulmonary edema and acute left-ventricular failure [1]. On the other hand, unilateral RAS can proceed without symptoms, especially in patients treated with drugs blocking the Renin–Angiotensin–Aldosterone system [2,3]. Significant RAS with clinical symptoms can be treated conservatively, and if that is insufficient, invasively, by percutaneous angioplasty with or without stent placement, or surgically. The most common cause of RAS is atherosclerotic renovascular disease (ARVD) [1,3]. Although first reports concerning renal artery revascularization were optimistic and showed the advantages of this invasive procedure in lowering blood pressure and improving kidney function, further randomized prospective studies did not confirm these findings [1]. One reason for these inconclusive results can come from different inclusion criteria or differences in the best revascularization timing. Moreover, operator experience, procedure technique, atherosclerosis advancement, quantity of stenoses, and adverse events such as atheroembolism cannot be ignored. On the other hand, the criteria for significant renal artery stenosis were not definitely established, depending on the diagnostic method and use of relative measures. The most popular criterion of significant RAS is the reduction in arterial lumen by over 50–60%. To better predict the benefits of invasive procedures, the latest recommendations for RAS treatment suggest using clinical as well as lesion criteria for revascularization [4]. The severity of RAS with a diameter reduction of 50–69% should be confirmed by additional hemodynamic alterations in a fractional flow reserve or systolic trans-lesional gradient (although their information adequacy about the hemodynamic significance of the stenosis is disputable), but stenoses with a ≥70% diameter reduction are thought to be severe enough, and additional tests are not expected [4,5]. Nevertheless, percentage reduction in area and diameter are relative measures. However, if an idea of measurement of the minimal diameter or area of the stenosis lumen is comprehensible, establishing the best reference point for normal, non-stenotic renal artery lumen is disputable and difficult, especially in ostial stenoses, which occur primarily in atherosclerotic etiology [6]. Thus, the results of renal artery stenosis measurement can differ among investigators. This discrepancy can result in improper qualifications for revascularization and its inconclusive advantages. Using the actually proposed same criterion of diameter or lumen reduction in renal arteries of different native dimensions can result in even a twofold difference in the area of the stenotic lumen and volume flow. However, this problem can be overcome by implementing stenosis criteria based on direct, non-relative measurements. This study aimed to investigate the renal artery stenosis parameters independently associated with renal blood flow estimated in contrast-enhanced multidetector computed tomography (CE-MDCT).

## 2. Materials and Methods

A total of 64 kidneys in 41 patients (22F + 19M, age 63.3 ± 15.9 years) with resistant hypertension diagnosed with atherosclerotic stenosis of supplying arteries were included in the study. Initially, an ultrasound Doppler examination of the renal arteries, with an assessment of intrarenal segmental arteries, and Color Doppler Dynamic Tissue Perfusion Measurement (DTPM) of the kidney cortex were performed; then, a low-dose CE-MDCT was conducted. Due to resistant hypertension, hypotensive medications were not withdrawn during imaging procedures. Inclusion criteria were age over 18 years, suspicion of renal artery stenosis in medical history, and written informed consent. Exclusion criteria comprise acute kidney injury, acute inflammation, kidney failure demanding renal replacement therapy, iodide contrast intolerance, accessory renal arteries occurrence, renal artery hypoplasia, or kidney agenesis and CE-MDCT artifacts, which made the proper stenosis assessment impossible. Informed consent was obtained from all patients included in the study.

### 2.1. Renal Function Assessment

Before kidney perfusion evaluation, based on creatinine from a fasting blood sample, the CKD–EPI formula was used to calculate the estimated glomerular filtration rate (eGFR) [7].

### 2.2. Contrast-Enhanced Multidetector Computed Tomography

The dynamic measurement of cortical blood flow in the three-dimensional region of interest (ROI) encompassing the renal cortex was performed based on contrast agent renal arteries and the dynamic measurement of cortical blood flow in the three-dimensional region of interest (ROI). During intravenous administration of an iso-osmolar contrast agent (Visipaque 320 mg/mL, GE Healthcare), the ROI was repeatedly scanned (single-source DECT scanner with rapid kVp switching, Discovery CT 750 HF; GE Healthcare, Waukesha, WI, USA). The readings of blood flow acquired in the selected ROI were normalized to those obtained in the aorta. To avoid artifacts, patients were asked for shallow and slow breathing. Scans were evaluated by one radiologist with over 20 years of experience in computed tomography.

#### 2.2.1. CE-MDCT Protocol

**Figure 1:**
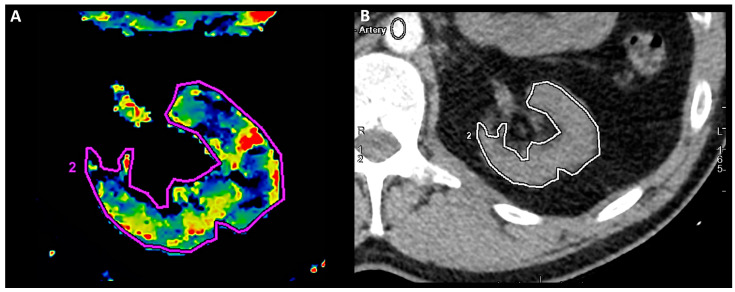
Left-kidney perfusion assessment. Manually outlined ROI (2) on transverse section of left kidney in CE-MDCT perfusion assessment (**A**) and in relation to other anatomical structures (**B**).

The examination consisted of two phases and was described earlier [8]. Briefly, the first phase comprised non-contrast helical abdomen scans for kidney localizing. The second phase concerned a 14 cm scan-area length, encompassing both kidneys and using intravenous infusion (4.0 mL/s) of a nonionic contrast medium. In every examination, 25 shuttle passes were performed, providing 375 images (tube voltage 100 keV, helical thickness 5 mm, rotation time 0.4 s). For cortical perfusion assessment, an ROI was manually outlined in the transverse section of the kidney on the level of the middle column and pelvis (Figure 1). The captured scans were investigated with Advantage Workstation server 4.7 (GE Healthcare, USA), equipped with software CT perfusion 4D, which automatically calculated the cortical blood flow (mL/100 g/min) corresponding to ROI perfusion.

#### 2.2.2. Renal Artery Stenosis Measurement

**Figure 2:**
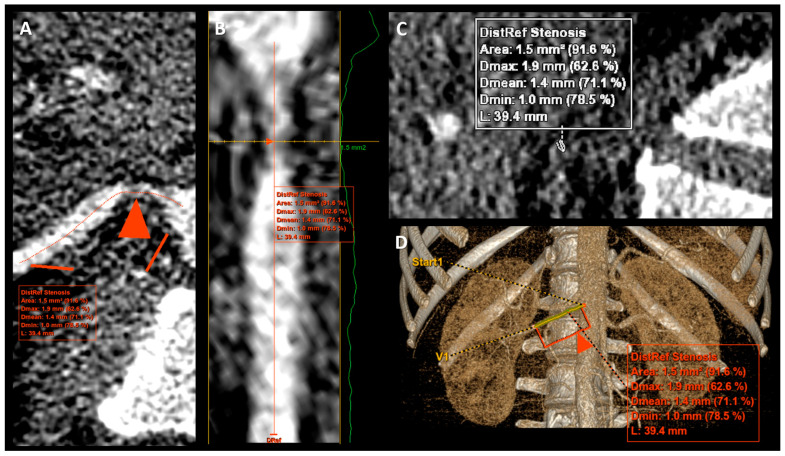
Reconstruction images with measurement of right renal artery stenosis. (**A**–**D**)—reconstruction images of the same right renal artery. The red triangle arrow (**A**,**B**,**D**) indicated a point with maximal stenosis.

Renal artery angiographic scans were reconstructed in 0.625 mm layers from II phase of perfusion assessment and contrast media infusion (Figure 2). Direct measurements of stenosis comprised minimal cross-sectional area (CSA), minimal diameter (MinD), and maximal diameter (MaxD) of lumen stenosis; then, mean diameter (MeD) was calculated according to the formula: MeD = (MinD + MaxD)/2. Due to the ostial and proximal atherosclerotic alterations in renal arteries, for relative parameters, the reference measurement point was set in the distal, non-stenotic part of the main renal artery, in an average 20–30 mm from the aortal ostium and 10 mm before the bifurcation [6]. Then, normal lumen CSA and normal diameter (NormD) were estimated. Relative parameters of stenosis were calculated as follows: maximal diameter reduction (MaxDR) [%] = 100 × (1 − MinD/NormD), and lumen cross-sectional area reduction (CSAR) [%] = 100 × (1 − minimal CSA/normal CSA).

### 2.3. Kidney Ultrasound Assessment

**Figure 3:**
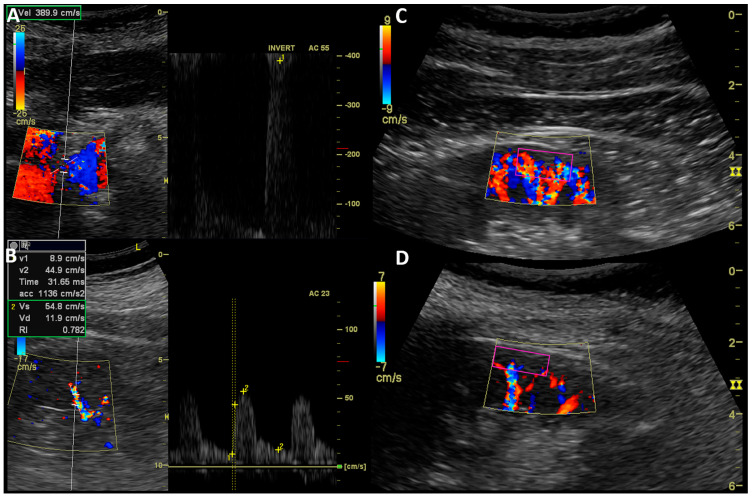
Ultrasound measurement of RAS and kidney perfusion. Doppler ultrasound examination: (**A**)—Measurement of peak systolic velocity in stenosis of right renal artery; (**B**)—Acceleration, acceleration time, and resistive index assessment in interlobar artery of left kidney; (**C**)—cortical perfusion assessment in ROI (purple rectangle) of kidney with insignificant RAS (CSAR 25%, MeD 6.5 mm); (**D**)—cortical perfusion assessment in ROI (purple rectangle) of kidney with significant RAS (CSAR 80%, MeD 3.5 mm).

Ultrasound examination (2–5 MHz curved array probe with Logiq P6, GE Healthcare, Seoul, Republic of Korea) of kidneys encompass the kidney length, hemodynamic Doppler-derived flow properties in renal arteries (PSV—peak systolic velocity [cm/s] and RAR—renal-to-aortic-PSV ratio), and intrarenal (ACC—acceleration [cm/s^2^], ACT—acceleration time [ms] and RI—resistive index [ratio]) as mean values of 2–3 readings performed in different segmental or interlobar arteries (Figure 3A,B). A dynamic tissue perfusion measurement (DTPM) method with an assessment of arterial flow through the selected ROI as a marker of renal cortex perfusion (RCP [mL/s]) was used [8,9]. An ultrasound color Doppler ROI was set in the middle cortical segment of the kidney in the longitudinal projection, between centers of two medullar pyramids and between the outer pyramids margin and renal capsule (Figure 3C,D). A color Doppler gain was constant and never changed. However, the scale velocity of the color Doppler option was adjusted to achieve the best visual flow mapping and to avoid artifacts. Short movie sequences lasting 3–5 s and recorded in DICOM standard, containing visualization of cortical flow, were transferred to an external PC software (PixelFlux v.18–03–11, Chameleon Software, Leipzig, Germany) to perform a semiautomatic clips analysis.

### 2.4. Statistical Analysis

The results were presented as a mean with standard deviation and median with interquartile range (IQR). Pearson’s or Spearman’s correlation test was used to test relations between the investigated variables according to the conformity with normal distribution, which was proved with the Shapiro–Wilk test. To visualize the relationship between the tested parameters, scatterplot diagrams with distance-weighted least squares fitting were drawn. The stepwise retrograde multivariable regression analysis was used to investigate independently related variables. Finally, the ROC analyses were performed to find the best cut-off points for investigated parameters. Only the two-sided *p* < 0.05 was considered significant. For statistical analysis, Tibco Statistica software v. 13.3 (StatSoft Polska Sp z o. o., Cracow, Poland) was used.

## 3. Results

Characteristics of the study group and results of investigated parameters are presented in Table 1. Mean eGFR indicated chronic kidney disease (CKD) in stage 3. However, the eGFR range (min. 9.7; max. 123 mL/min/1.73 m^2^) shows a full spectrum of kidney function (CKD stages 1–5). Moreover, a mean CSAR of 55.3% suggested a near-significant stenosis of the investigated renal arteries, but the range of the studied CSAR was quite wide and encompassed stenoses between 7.7% and 98.0%.

The results of the correlation analysis between the estimated parameters and considered RAS measures are shown in Table 2.

The renal blood flow estimated in the CE-MDCT correlated significantly with age (r = −0.371; *p* = 0.003), creatinine (r = −0.425; *p* < 0.001), and CKD–EPI (r = 0.491; *p* < 0.001), whereas dRCP was similarly substantially associated with age (r = −0.355; *p* = 0.005), creatinine (r = −0.395; *p* = 0.002), CKD–EPI (r = 0.428; *p* < 0.001), and CBF (r = 0.413; *p* = 0.001).

In the stepwise retrograde multivariable regression analysis model, including the parameters of stenosis measurements (CSAR, MeD, MinD) that significantly correlated with CBF, only the mean stenosis diameter was independently associated with CBF (R^2^ = 0.179; *p* < 0.001).

**Figure 4:**
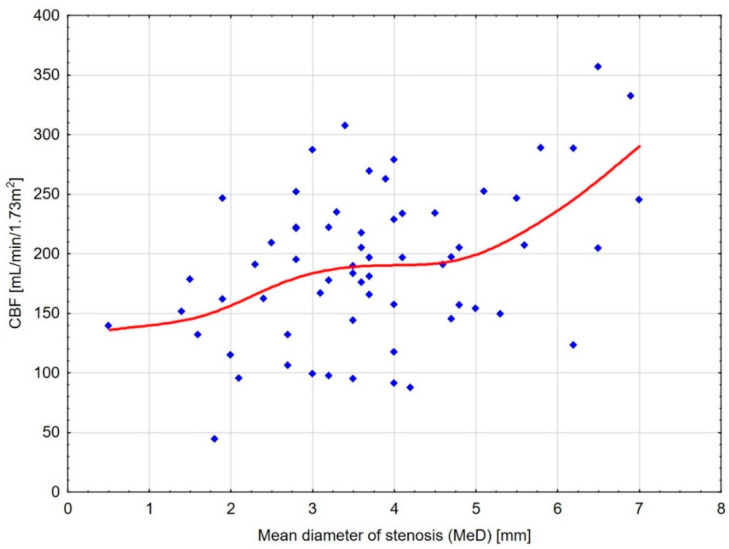
Scatterplot diagram for CBF and MeD with distance-weighted least squares fitting. The diagram shows a substantial decrease in kidney cortical blood flow up to the mean diameter of stenosis (MeD) of about 4.5 mm and its subsequent reduction starting from 3.5 mm.

**Figure 5:**
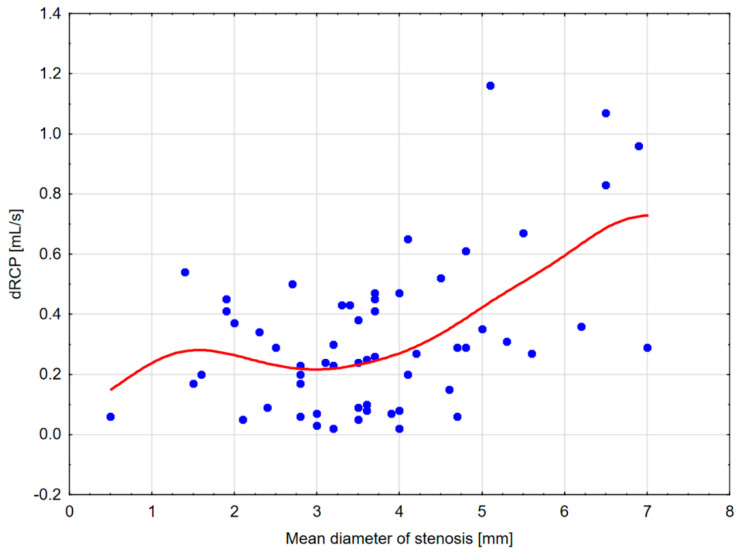
Scatterplot diagram for dRCP and MeD with distance-weighted least squares fitting. The diagram shows a substantial decrease in kidney cortical perfusion measured with the DTPM method up to the mean stenosis (MeD) diameter of about 3–3.5 mm.

To show the possible cut-off point values for lowering MeD, for which the decrease in kidney perfusion becomes substantial, scatterplot diagrams were drawn (Figure 4 and Figure 5), and ROC analyses for different CBF thresholds were performed (Table 3).

An analysis of the scatterplots showed a substantial decrease in CBF up to an MeD of about 4.5 mm and a further subsequent reduction beginning from 3.5 mm. In addition, a dRCP decrease was observed up to MeD of 3–3.5 mm. Moreover, the reduction in CBF ≤ 150–175 mL/100 g/min corresponded with an MeD of ≤3.5 mm (Table 3). Former analyses of other measurement thresholds corresponding to MeD ≤ 3.5 mm were shown in Table 4.

## 4. Discussion

In the presented work, we show a better correlation of the direct stenosis measures in the form of minimal stenosis diameter (MinD) and mean stenosis diameter (MeD) with renal cortex perfusion, in comparison to relative parameters, such as lumen cross-sectional area reduction (CSAR) and maximal diameter reduction (MaxDR). The observations were similar in an objective CE-MDCT perfusion estimation and in the more operator-dependent dynamic color Doppler ultrasound examination.

Our results are in line with a previous study by Andersson et al., who investigated 68 kidneys in 47 patients with clinical suspicion of RAS in both computed tomography and magnetic resonance angiography, then verified these results with captopril-enhanced renography and captopril test, which were positive in 11 cases [6]. The authors found that direct measures of stenosis had the highest area under ROC if the stenosis was in the proximal part of the renal artery, while relative measures were better in renal arteries with the largest maximal diameter of the unchanged part. However, these differences were not significant. This suggests that direct and relative parameters are almost equally important. However, atherosclerotic RAS occurred mainly in the ostium and proximal part of renal arteries. Moreover, in the abovementioned study, stenoses of accessory arteries were also analyzed, and the influence of investigated measures was not correlated with renal cortical blood flow.

The RAS diagnosis and treatment guidelines consider renal artery disease if the arterial lumen is reduced by ≥60% or if the diameter is reduced by ≥50%, which are relative measures calculated from the ratio of maximal stenosis dimensions to measures of the non-changed artery lumen [3]. However, in non-stenotic arteries, the diameter and vascular lumen change along the vessel [10]. In addition, post-stenotic lumen dilatation can lead to stenosis overestimation in relative measures [6]. Therefore, measurement of the normal lumen in other fragments of unchanged renal artery is burdened with unexpected errors. Moreover, the lumen of the normal renal artery can significantly differ in diameter, ranging from 4.2 ± 1.1 mm to 7.1 ± 1.6 mm, including differences between the left and right renal arteries. This diversification enabled different abilities for kidney blood supply, which sometimes exceeds 100% and persists; although, the same lumen stenoses in different arteries are recognized. This could produce essential errors in quantifying RAS and influence the effectiveness of the revascularization procedure. Moreover, this phenomenon could be associated with higher cardiovascular risk even if low-grade RAS occurred and consideration to revascularize clinically but not hemodynamically significant RAS < 50% [5].

Available case reports concerning invasive treatment of renal artery stenosis showed the advantages of percutaneous angioplasty in lowering blood pressure and improving kidney function [11,12]. The meta-analysis of Leertouwer et al., which included 678 patients (24 studies) with renal artery revascularization with stent placement, proved clinical improvement in hypertension in about 70% of patients, and renal function improvement occurred in 30% of patients [13]. However, randomized clinical trials were not so conclusive. In the STAR trial (Stent Placement for Atherosclerotic Stenosis of Renal Artery), patients with RAS-only ≥50% and eGFR < 80 mL/min/1.73 m^2^ were qualified for angioplasty and medical treatment or medical treatment alone and found no advantage from invasive treatment in slowing CKD progression [14]. However, about 30% of patients from the intervention group had RAS < 50%. The ASTRAL (Angioplasty and Stenting for Renal Artery Lesions) trial, which included 806 patients (60% with RAS > 70% estimated only in ultrasound examination and not verified with objective imaging method), similarly reported no benefit of invasive procedure over medical therapy alone with regard to blood pressure improvement and renal function decline or mortality [15]. Although the first results of CORAL (Cardiovascular Outcomes for Renal Atherosclerotic Lesions) trial included 931 patients with RAS (mean stenosis of 67% in angiography) showed no cardiovascular and renal outcome benefit, it was changed in a post hoc analysis performed after five years of follow-up, which showed improved overall survival and event-free survival from the composite end-point consisting of cardiovascular death and progression of chronic kidney disease in patients with initially elevated albuminuria [16,17]. On the other hand, in low-grade (30–50%) unilateral RAS, renal plasma clearance of asymmetric dimethylarginine (ADMA) is significantly lower in comparison to non-stenotic contralateral kidney, and this difference rises in unilateral RAS ≥ 50% [5]. The abovementioned study suggests the functional significance of hemodynamically insignificant RAS defined by relative criteria, leading to differences in oxidative stress and fibrosis between contralateral kidneys [18]. An analysis of discrepancies in the results of presented clinical trials questioned the pathophysiological concept and the method of patient selection [5]. These data suggest that improper qualification for invasive renal artery revascularization (insignificant or poorly verified RAS) contributed to the lack of expected cardiovascular and renal benefits. In addition, complications of renal artery revascularization, such as atheroembolism, indicated in 2.9–77% of cases, and contrast medium-induced nephropathy could substantially influence potential benefits of this invasive treatment; therefore, an estimation of the post-procedure percentage change in kidney function can help in proper effect assessment of these events [19,20].

Although the same degree of RAS is recognized in arteries of various diameters, different amounts of kidney blood supply can result in effects ranging from slight, almost normal restriction of organ perfusion (>200 mL/100 g/min), to its significant ischemia (<150 mL/100 g/min). This could implicate radically different levels of pressor pathways activation (renin increase, afferent sympathetic nerve activation and hypertension, tissue hypoxia, inflammation, injury, and fibrosis), resulting in different outcomes of invasive RAS treatment [1]. The precise stenosis evaluation, and thereby blood supply restriction, seems to be critical for the investigated lack of alterations in the intrarenal oxygenation of an up to 30–40% decrease in the renal blood flow, which is probably a result of the renal blood flow exceeding the metabolic demand of renal tissue in normal conditions but is necessary for glomerular filtration rate preservation [21]. This is consistent with our results, which showed an almost proportional decline in renal cortical perfusion with decreasing MeD up to 4.5 mm for CBF and 3.5 mm for dRCP. Moreover, both parameters of blood flow, CBF, and dRCP were correlated with kidney function. In the study of 48 kidneys with atherosclerotic renovascular disease, Abdelrhman et al. showed stable kidney cortex oxygenation in decreasing renal blood flow up to 200 mL/min and rising kidney hypoxia with a further flow decrease [21]. This flow threshold likely corresponds to the MeD < 3.5–3.6 mm (CBF < 175–200 mL/100 g/min) estimated in our study. Moreover, the recent statement for RAS revascularization suggests that the occurrence of kidney tissue hypoxia is the best moment for invasive treatment to achieve maximal clinical benefit [1]. Considering the abovementioned information and our results, it seems that the MeD reduction between 3.5 and 3.6 mm when the CBF decreases below 175–200 mL/100 g/min could be proposed as the best stenosis frame for revascularization.

Due to different methods of blood flow estimation, the threshold for proper and decreased CBF is considered between 150 and 200 mL/100 g/min, which corresponds with the MeD between 3.5 and 3.6 mm. In the study by Li et al., including 33 diabetic patients with CKD (eGFR 50.0 ± 13.9 mL/min/1.73 m^2^) and 30 healthy controls examined with arterial-spin-labeling magnetic resonance, the renal cortical blood flow in healthy controls (207.3 ±41.8) was significantly higher than in the CKD group (108.4 ± 36.4), and a threshold cortical perfusion value of 142.9 [mL/100 g/min] significantly separated the investigated groups (AUC, 0.98; sensitivity, 84.9%; specificity, 100.0%) [22]. In another study, renal cortical perfusion differentiated a group of 30 healthy volunteers (302.6 ± 49.9 mL/100 g/min) from patients with glomerulonephritis with preserved and decreased kidney function (*n* = 11, eGFR = 106.2 ± 26.2 with CBF = 247.9 ± 34.9 vs. n = 13, eGFR = 25.1 ± 16.6 mL/min/1.73 m^2^ with CBF = 172.8 ± 42.1 mL/100 g/min) [23]. Based on contrast-enhanced computed tomography, proper renal cortical perfusion can fluctuate from 281.5 to 323.8 mL/100 g/min [24]. An investigation performed in the non-stenotic kidneys of patients with CKD (eGFR = 58.3 ± 28.1 mL/min/1.73 m^2^) revealed mean CBF = 247.9 ±98.6 mL/100 g/min, which significantly correlated with eGFR (r = 0.606, *p* < 0.001) [8].

We used the dynamic tissue perfusion measurement method for renal cortex perfusion assessment. This ultrasound semiautomatic technique was helpful, among others, in diagnosing cardiorenal syndrome and differentiating between glomerulonephritis and hypertensive nephropathy [25,26]. However, the threshold value of dRCP differentiating the proper and decreased kidney function has not yet been established. Recently published investigations performed in patients with hypertension and comparable kidney dysfunction (mean eGFR 58.3 ± 28.1 vs. 52.8 ± 25.6 mL/min/1.73 m^2^) showed substantially higher dRCP than in presented patients with RAS (0.303 vs. 0.211 cm/s) [8]. Moreover, in the Gutowski et al. study, patients with severe COVID-19 were included, and dRCP was the independent marker associated with the oxygenation ratio expressing disease severity [27]. In patients with CKD, those with suspected hypertensive nephropathy had slightly lower dRCP than those with glomerulonephritis (0.289 vs. 0.346 mL/s; *p* = 0.052) [26]. These values were comparable to the mean dRCP in our patients (0.357 mL/s), but for MeD = 3.5 mm, dRCP decreases to 0.250 mL/s, which seems substantially less.

Although the presented results are promising, our study had some limitations. First, the evaluation of the renal arteries was based on image reconstruction from time-frames of 5 mm, which were captured in time of cortical blood flow assessment. Thus, thinner 2–3 mm layers could be more precise and strengthen the significance of the direct stenosis measurement. On the other hand, the quality of acquired images allows reconstruction to 0.625 mm layers, which partially overcomes this inaccuracy. Second, as for RAS measurement, the group of investigated patients seems limited, which influences the lower significance of presented results. On the other hand, we examined one of the largest groups considering the evaluation of renal cortex blood flow [22,23,28]. Moreover, due to the lack of observations, we could not verify our results with the clinical benefits of revascularization in the proposed range of mean stenosis diameter, which makes these advantages probable but unconfirmed. Thus, further studies on larger patient groups treated with RAS revascularization should confirm our results. In addition, we believe that using CE-MDCT made our investigations sufficiently detailed and robust, which enabled the confirmation of results with ultrasound methods. On the other hand, we did not compare different imaging methods, such as arteriography and magnetic resonance. Moreover, we presented the results of the retrospective research; therefore, inclusion biases cannot be excluded. A prospective study validating our findings and using different imaging methods could confirm the applicability of the results in clinical settings. Nevertheless, using two independent imaging methods, we confirmed the substantial decline in the renal cortical blood flow associated with the mean stenosis diameter reduction, with a nadir value of 3.5 mm.

## 5. Conclusions

Renal cortical perfusion estimated in both the dynamic ultrasound tissue perfusion measurement and contrast-enhanced multidetector computer tomography better correlates with absolute than relative renal artery stenosis measures. The directly measured mean diameter of renal artery stenosis is independently associated with renal cortical blood flow. It is probably a more appropriate and less error-burdened parameter for the invasive RAS treatment criterion. However, these findings should be confirmed in controlled clinical trials due to limited data and lack of prospective observations.

## Figures and Tables

**Table 1:** Characteristics of the study group and results of the investigated parameters.

Parameter	Mean	SD	Median	IQR
Age [years]	61.8	15.0	65.1	18.3
Creatinine [mg/dL]	1.62	0.94	1.30	0.80
eGFR [mL/min/1.73 m^2^]	52.8	25.6	47.0	30.8
Kidney lenght [mm]	109.4	41.8	107.5	19.5
CSAR [%]	55.3	21.4	55.0	30.0
MeD [mm]	3.74	1.42	3.60	1.85
MinD [mm]	2.81	1.23	2.80	1.20
MaxDR [%]	41.19	18.69	37.00	22.50
PSV [cm/s]	199.1	95.4	185.0	129.5
RAR	2.39	1.46	2.00	1.98
ACC [m/s^2^]	6.61	4.16	6.00	4.79
ACT [ms]	46.6	24.7	39.7	22.0
RI [ratio]	0.703	0.104	0.735	0.155
CBF [mL/100 g/min]	190.7	64.5	191.0	86.7
dRCP [mL/s]	0.357	0.398	0.280	0.345

ACC—acceleration; ACT—acceleration time; CBF—cortical blood flow; Creatinine—serum creatinine concentration; CSAR—lumen cross-sectional area reduction in stenosis; dRCP—renal arterial perfusion in DTPM method; eGFR—estimated glomerular filtration rate based on CKD—EPI formula; MinD—minimal diameter of stenosis; MeD—mean diameter of stenosis; MaxDR—maximal diameter reduction in stenosis; PSV—peak systolic velocity in the renal artery; RAR—renal to aortic velocity ratio; RI—renal resistive index.

**Table 2:** Results of correlation analysis—correlation coefficients with significance levels.

Parameter	CSAR [%] (p)	MeD [mm] (p)	MinD [mm] (p)	MaxDR [%] (p)
Age [years]	0.307 (0.014)	−0.340 (0.006)	−0.133 (0.293)	0.167 (0.186)
Creatinine [mg/dL]	0.232 (0.066)	−0.116 (0.363)	−0.201 (0.111)	0.212 (0.093)
eGFR [mL/min/1.73 m^2^]	−0.220 (0.081)	0.196 (0.120)	0.246 (0.049)	−0.206 (0.102)
Kidney length [mm]	−0.098 (0.444)	0.233 (0.067)	0.345 (0.011)	−0.035 (0.785)
PSV [cm/s]	0.252 (0.052)	−0.346 (0.007)	−0.448 (<0.001)	0.336 (0.009)
RAR	0.215 (0.099)	−0.283 (0.028)	−0.351 (0.006)	0.305 (0.018)
ACC [m/s^2^]	0.067 (0.599)	0.039 (0.760)	0.084 (0.510)	0.173 (0.176)
ACT [ms]	−0.076 (0.556)	−0.047 (0.712)	−0.113 (0.379)	−0.065 (0.614)
RI [ratio]	−0.007 (0.958)	−0.105 (0.414)	−0.027 (0.833)	0.013 (0.922)
CBF [mL/100 g/min]	−0.422 (0.003)	0.344 (0.005)	0.348 (0.005)	−0.190 (0.133)
dRCP [mL/s]	−0.167 (0.201)	0.357 (0.005)	0.427 (<0.001)	−0.150 (0.250)

ACC—acceleration; ACT—acceleration time; CBF—cortical blood flow in CE-MDCT; dRCP—renal arterial perfusion in DTPM method; Creatinine—serum creatinine concentration; eGFR—estimated glomerular filtration rate based on CKD–EPI formula; PSV—peak systolic velocity in the renal artery; RAR—renal artery to aortic velocity ratio; RI—renal resistive index.

**Table 3:** Results of ROC analyses for mean diameter of stenosis and different renal CBF thresholds.

CBF Threshold [mL/100 g/min]	MeD Cut-Off [mm]	Sensitivity [%]	Specificity [%]	AUC	*p*-Value
100	3.4	57.1	61.4	0.639	0.134
150	3.5	64.7	59.6	0.634	0.099
175	3.5	60.0	61.5	0.649	0.038
200	3.6	59.5	59.3	0.681	0.007
250	3.7	62.3	63.6	0.697	0.021
300	4.0	68.9	66.7	0.790	0.067

AUC—area under curve; CBF—cortical blood flow in CE-MDCT; MeD—mean diameter of stenosis.

**Table 4:** ROC analysis for mean diameter of stenosis at 3.5 mm.

Variable	Cut-Off Value	Sensitivity [%]	Specificity [%]	AUC	*p*-Value
CSAR [%]	58.0	73.3	73.5	0.734	<0.001
MinD [mm]	2.6	90.0	88.2	0.944	<0.001
MaxDR [%]	37.8	63.3	67.6	0.668	0.005
CBF [mL/100 g/min]	189.8	63.3	64.7	0.653	0.027
dRCP [mL/s]	0.250	71.9	60.7	0.662	0.020
eGFR [mL/min/1.73 m^2^]	46.3	56.7	55.9	0.567	0.359

## Data Availability

The dataset is with the authors and available in scientific interest on request.
